# Will we ever eradicate animal tuberculosis?

**DOI:** 10.1186/s13620-023-00254-9

**Published:** 2023-09-22

**Authors:** Christian Gortázar, José de la Fuente, Alberto Perelló, Lucas Domínguez

**Affiliations:** 1 SaBio Instituto de Investigación en Recursos Cinegéticos IREC (UCLM & CSIC), Ciudad Real, Spain; 2 Department of Veterinary Pathobiology, Center for Veterinary Health Sciences, Oklahoma State University, Stillwater, OK USA; 3 Sabiotec, Camino de Moledores s/n. 13003, Ciudad Real, 13071 Spain; 4 VISAVET and Department of Animal Health-Faculty of Veterinary Medicine, Universidad Complutense Madrid, Madrid, Spain

**Keywords:** Acceptability of control options, Farm biosafety, Integrated disease control, Maintenance host community, Test and cull, Vaccination

## Abstract

Two characteristics of the *Mycobacterium tuberculosis* complex (MTC) are particularly relevant for tuberculosis (TB) epidemiology and control, namely the ability of this group of pathogens to survive in the environment and thereby facilitate indirect transmission via water or feed, and the capacity to infect multiple host species including human beings, cattle, wildlife, and domestic animals other than cattle. As a consequence, rather than keeping the focus on certain animal species regarded as maintenance hosts, we postulate that it is time to think of complex and dynamic multi-host MTC maintenance communities where several wild and domestic species and the environment contribute to pathogen maintenance. Regarding the global situation of animal TB, many industrialized countries have reached the Officially Tuberculosis Free status. However, infection of cattle with *M. bovis* still occurs in most countries around the world. In low- and middle-income countries, human and animal TB infection is endemic and bovine TB control programs are often not implemented because standard TB control through testing and culling, movement control and slaughterhouse inspection is too expensive or ethically unacceptable. In facing increasingly complex epidemiological scenarios, modern integrated disease control should rely on three main pillars: (1) a close involvement of farmers including collaborative decision making, (2) expanding the surveillance and control targets to all three host categories, the environment, and their interactions, and (3) setting up new control schemes or upgrading established ones switching from single tool test and cull approaches to integrated ones including farm biosafety and vaccination.

## Introduction

Animal tuberculosis (animal TB) is the disease caused by infection of wild and domestic animals with *Mycobacterium bovis* and closely related members of the *Mycobacterium tuberculosis* complex (MTC). The infection of human beings with MTC members other than *M. tuberculosis* is known as zoonotic TB. Despite enormous efforts and costs, complete eradication of the causative agents of animal TB has so far only been achieved in Australia, a country with no relevant non-bovine MTC maintenance hosts [1, 2].

This overview proposes a critical look into the future of animal TB control. The first section addresses the epidemiology of animal TB with a focus on several important but still neglected aspects such as the multi-host nature of the pathogen, pathogen genetic diversity, and its frequent indirect transmission. The second one addresses emerging tools with a significant impact on understanding TB epidemiology. The third section briefly describes the global situation of animal TB from a one health perspective, i.e., not only in cattle, but also in wildlife and in domestic animals different from cattle which are often neglected regarding TB control. The fourth and final section deals with the tools available for TB control in cattle and in other hosts and the opportunities to upgrade the current TB control strategies based on better stakeholder involvement and the concept of integrated disease control.

## Diversity in the host species, genome and transmission in animal tuberculosis

Two characteristics of the MTC are extremely relevant for animal TB epidemiology and control, namely their ability to survive in the environment and their capacity to infect multiple host species. Regarding the former, mycobacteria evolved from, and many species are, environmental organisms. Even the species which evolved to adapt to mammalian hosts, such as the members of the MTC, are still able to survive in the environment [3–6]. This means that one characteristic of *M. bovis* is the ability for indirect transmission mediated by substrates such as water or feed.

This has obvious implications for farm biosafety and preventive medicine, for instance, by considering feed- or water-mediated cross species transmission [4, 7], but also for the slaughterhouse inspection of cattle, since gastrointestinal transmission has been shown to produce different lesion severity and distribution both after experimental infections [8] and in natural settings [9].

Regarding the latter, some mycobacteria became specialized in parasitizing mammals, and so the MTC evolved, including *M. bovis*, possibly the MTC member with the broadest host range [10, 11]. *Mycobacterium bovis* can infect several different wild and domestic host species, and the more hosts are part of a given system, the more likely it is that TB is maintained through time in this system [12]. Furthermore, infections with members of the MTC are generally chronic and debilitating, leading to prolonged opportunities for transmission and for contaminating the environment [13].

**Fig. 1:**
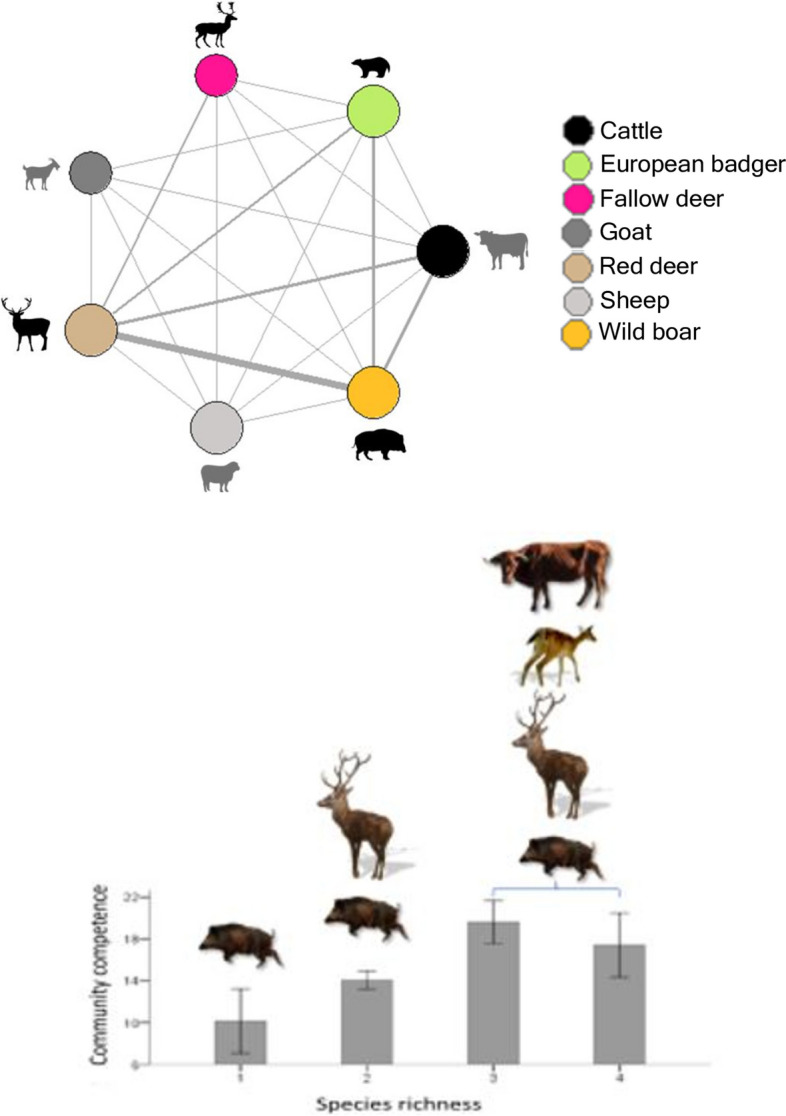
The maintenance community concept. The upper panel represents 7 *Mycobacterium tuberculosis* complex (MTC) host species including cattle, domestic animals other than cattle (sheep, goat), and wildlife (red and fallow deer, wild boar, badger) and their space-time coincidence (represented by the line thickness) in the Iberian Peninsula, as assessed through camera trap networks. The lower panel shows how the competence of a host community to maintain MTC circulation over time is larger in multi-host systems (at higher host species richness) than in single- or two-host ones. (modified from references [6] and [9])

In fact, it is time to forget the classical single or two-host system views, where only certain species were regarded as maintenance hosts [14] and think of complex and dynamic multi-host MTC maintenance communities where several different wild and domestic species and the environment contribute to build a network that facilitates pathogen survival (Fig. 1). The successful New Zealand example of progressive *M. bovis* eradication targets an almost complete host community including cattle, wildlife (brushtail possums, *Trichosurus vulpecula*) and domestic animals other than cattle (farmed deer) and is now aiming at TB freedom in cattle and farmed deer by 2026, TB freedom in possums by 2040, and biological eradication of *Mycobacterium bovis* by 2055.

Another aspect relevant to pathogen transmission is associated with its genetic diversity and the diverse species-specific host responses to infection. Regarding the human pathogen *M. tuberculosis*, genetic heterogeneity includes region- and host-specific variations that may affect pathogen transmission, prevalence, susceptibility to drug treatments and vaccine development [15–18]. Some species or clones of the MTC demonstrate extraordinarily little virulence for certain hosts, such as European badgers (*Meles meles*) for instance. In some populations, most of the infected badgers have no visible lesions and appear apparently healthy individuals [19]. By contrast, infected Eurasian wild boar (*Sus scofa*) are more likely to develop generalized TB in Mediterranean Iberia than in Atlantic habitats [20]. Among the possible explanations, it could be that the severe drought periods characteristic of the Mediterranean summer contribute to trigger clinical TB through an effect on host condition and immunity, through increased exposure and reinfection at waterholes, or through a combination of both mechanisms [21–23]. Infections with no significant lesion development (non-visible lesions, NVL) can last for long periods of time (years) but may eventually revert to clinical disease leading to pathogen shedding as soon as there is a change in the immune status of the infected individual [13, 19]. Whatever the cause, variability in both pathogens and hosts represents an additional challenge for TB control [24].

## Emerging technologies and their impact on TB epidemiology and control

Two new technologies already contribute significantly to our understanding of indirect MTC transmission, pathogen genetic diversity, and complex host and pathogen networks: whole genome sequencing (WGS) and environmental DNA. The first one, WGS, is increasingly used to establish the source of outbreaks and understand how infection spreads, i.e., the directionality of transmission [25]. It also allows deep insights into the phylogenetics and evolution of MTC members and the possible mechanisms of local adaptation, pathogenicity, and protective immunity [26]. Alternatively, tracking different species with GPS devices or camera trapping can be helpful in identifying risk hotspots and quantifying cross-species interactions in complex host communities [27–30].

Furthermore, the presence of MTC in the environment opens the possibility of non-invasive MTC monitoring and e-DNA-based risk analyses [31]. Environmental DNA has been instrumental for understanding MTC shedding patterns [6] and identifying environmental risk hotspots [4, 32]. Simple e-DNA technology such as sponge sampling has the potential to significantly contribute to on-farm risk identification and risk-mitigation [31, 33]. This technology shows early promise and might soon become deployable as a field tool.

Other new technologies ranging from selective breeding [34] to artificial intelligence (AI) and machine learning (ML) also have the potential to contribute to the future animal TB control [35, 36]. However, much needed breakthroughs in TB diagnosis are still pending, meaning that in-vivo TB diagnosis in all host species still relies on methods with a relatively poor sensitivity and variable specificity [24].

## A global perspective on animal tuberculosis

**Fig. 2:**
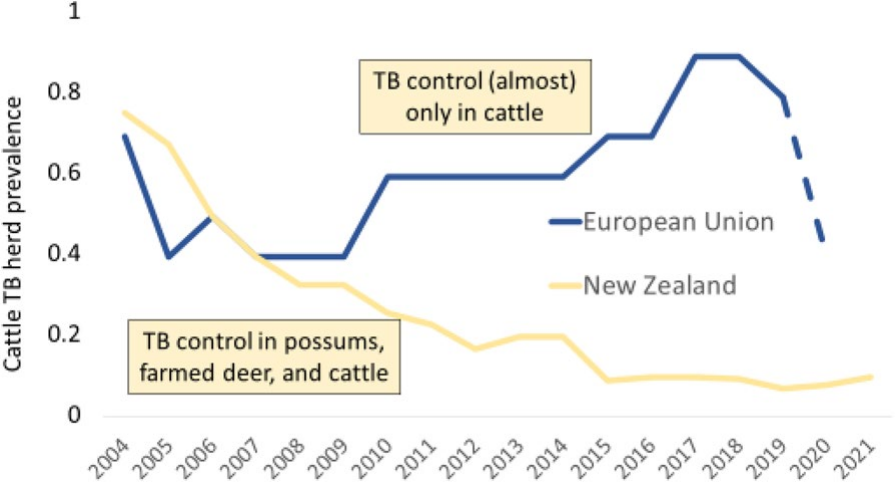
Mean annual cattle tuberculosis herd prevalence in the European Union (EU, blue) and in New Zealand (yellow) from 2004 to 2021. The dashed line indicates EU data excluding the UK after Brexit. Control targets all three host categories in New Zealand but almost only cattle in the EU

Animal TB represents a global challenge to health and economy [37]. Successful animal TB control, up to the level of being declared Officially Tuberculosis Free (OTF), has been achieved in many industrialized countries including the USA and Canada in North America, most members of the European Union, and Australia, among others. However, infection of cattle with *M. bovis* is reported in 44% of 188 WOAH territories from January 2017 to June 2018 [38] and is likely to occur in most countries around the world, including most OTF ones. In many of these, cattle TB occurs at very low prevalence due to the implementation of control or eradication programs over long periods of time [39]. In the UK, current bovine TB (bTB) herd prevalence levels of 6–14% are below the estimated 20–40% prevalence pre-compulsory controls in the 1940s-50s but still far away from eradication [39]. In the EU, the Member States are responsible for the eradication of bTB and may receive community financial support for the eradication program. However, funding for bTB control schemes is being phased out in EU. To meet the funding criteria, Member States must state eradication of bovine TB as the final target of their program [40]. However, complete eradication of bTB is not only difficult to achieve in high prevalence countries, but also in countries with low proportions of TB-infected herds including some OTF countries where TB prevalence is currently rising [41]. Figure 2 compares the time trends in cattle TB herd prevalence in the EU and in New Zealand.

**Fig. 3:**
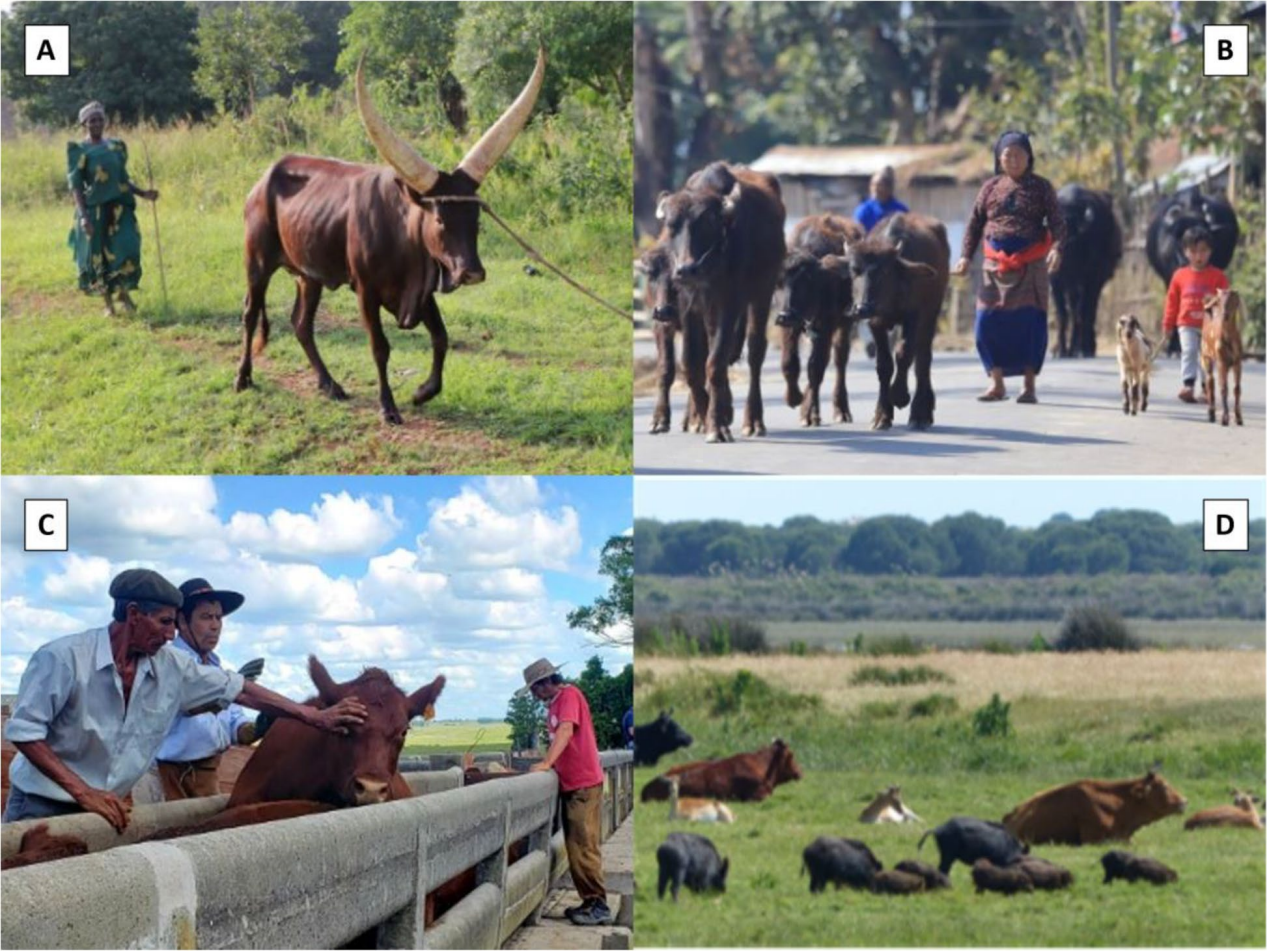
Challenges for global animal tuberculosis eradication. **A** In many low- and middle-income countries (LMIC), culling as a means of disease control is unacceptable for economic or religious reasons. **B** The role of domestic animals other than cattle in infection maintenance is unknown and often neglected, both in LMIC and in industrialized countries. **C** Many countries lack sufficient knowledge on the *Mycobacterium tuberculosis* complex maintenance community. This gap hinders improvements in tuberculosis control by targeting the whole host community. **D** Close interactions between host categories evidence the need to address all relevant hosts at the same time for successful tuberculosis control

In contrast with industrialized countries, in developing low- and middle-income countries (LMIC) where human and animal TB infection is endemic and bovine TB control programs are not implemented, the situation is like that described at the beginning of the 20th century when one out of nine human deaths were due to TB and 10–20% of them had an animal origin [42, 43]. In Uganda, 7.5% of the annual 559/100,000 pop total TB cases are due to zoonotic TB [44] and the cattle herd prevalence is as high as 28% [45]. In Nepal, young women who are more involved in handling with livestock or milking are more likely to suffer extrapulmonary TB [46], and 18% of cattle and 32% of water buffalo test TB positive [41]. In LMICs, standard TB control through testing and culling, movement control and slaughterhouse inspection are often too expensive or just unacceptable due to economic or religious reasons, and the TB situation in domestic animals other than cattle remains largely unexplored (Fig. 3). Considering that for many LMICs there are bigger challenges regarding animal health, vaccination emerges as the only potentially acceptable intervention.

One major complicating factor preventing animal TB eradication is its occurrence in wild mammal species [39]. This is the case of white-tailed deer (*Odocoileus virginianus*) in Michigan, the brushtail possum in New Zealand, or different wildlife hosts in several parts of Europe. In many other regions worldwide, the role of wildlife MTC maintenance hosts, or even their very existence, remains unexplored [47]. Even worse, while it is well established that several domestic animals other than cattle such as sheep, goats, pigs, or camelids can contribute to MTC maintenance, the situation of TB in domestic hosts other than cattle remains largely unknown in most regions of the world. The World Bank (2011; https://documents1.worldbank.org/curated/en/323671468179364909/pdf/668590WP00PUBL00Livestock0Atlas0web.pdf) listed TB among the top ten most important animal diseases not only in cattle but also in water buffalo, pigs, and camelids – a clear hint of the global relevance of domestic animals other than cattle for MTC maintenance. This is critical because in certain regions it has been estimated that domestic animals other than cattle represent about 50% of the total infected animals, and that the sum of infected wildlife and infected domestic hosts other than cattle completely outnumbers infected cattle [48].

## The way forward: from single tool to integrated disease control

As stated above, MTC is likely to persist in complex multi-host communities rather than in relatively simple one- or two-host systems. However, knowing which hosts are most relevant in a specific system as well as their abundance, distribution and interaction is not easy. In Ireland for instance, estimating the badger population size was highly challenging [49] and revealed a rather small number (63,188; 5–95th percentile 48,037–79,315) as compared to 7.3 million cattle, 5.5 million sheep, and 1.6 million pigs. The Irish TB host system might be locally more complex and include sika deer (*Cervus nippon*), too [25, 50]. In Argentina, open-air domestic pigs and invasive feral pigs (wild suids) contribute to MTC maintenance along with cattle [51] and the host community might also include other wildlife [52]. Thus, the first requirement when faced an endemic animal TB situation is running an epidemiological diagnosis, i.e., describing which species, both domestic and wild, are locally involved in MTC maintenance, their numbers, and how they are connected to each other [31].

Over time, certain regions repeatedly show high cattle TB incidences and allocation of more resources to these areas may be needed to combat the disease [53]. Alternatively, problem regions might hypothetically be excluded from the general control program or granted certain exceptions to cope with particularly challenging risk factors. Both options would imply a geographical zoning of disease control.

Beyond zoning, the classic control measures for cattle TB control are diagnostic testing and slaughter of test-positive animals, combined with movement control of infected herds and post-mortem inspection of cattle at the abattoir for presence of typical tissue lesions [39, 54]. Age culling was reportedly one of the ingredients for the successful eradication in Australia [1] but is not commonly listed among the TB control tools in cattle.

**Fig. 4:**
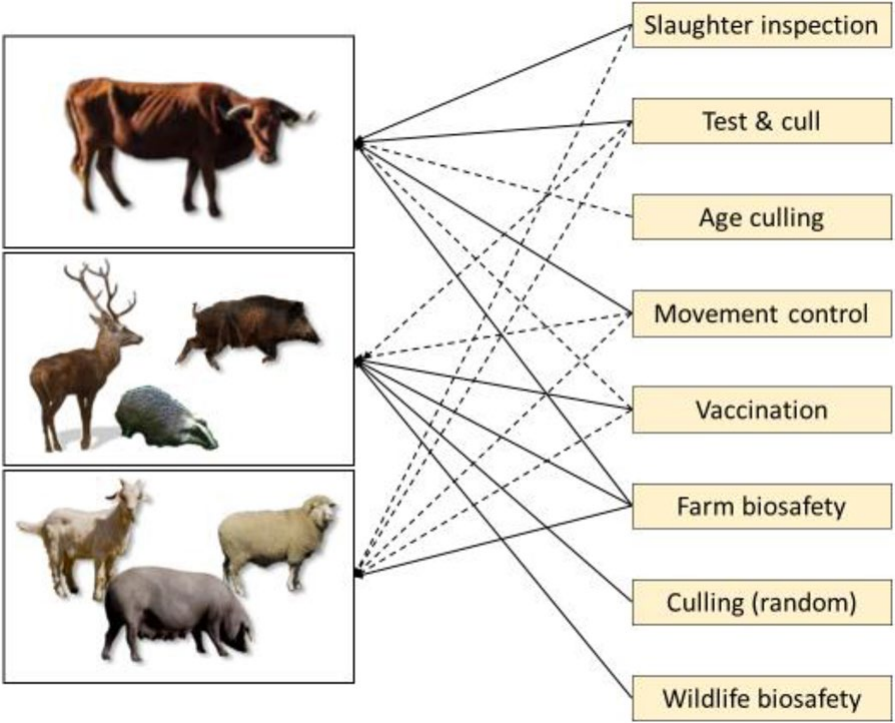
Animal tuberculosis control tools. Integrated schemes should consider all three host categories, cattle, domestic animals other than cattle, and wildlife (left). Intervention options (right) should ideally be combined and target more than one host category whenever possible. Solid lines indicate regular use (in some countries), dashed lines indicate possible or occasional use

In wildlife, the options for dealing with TB are generally limited to segregation of domestic animals from wildlife, which is targeting cattle TB control rather than wildlife TB (farm biosafety; [33]), wildlife-oriented biosafety and hygiene (e.g., safe carcass disposal; [55]), random or targeted culling of the wildlife hosts [56, 57], or vaccination [58]. Innovative and promising combined approaches include a badger test and vaccinate or remove trial in Northern Ireland [57]. The third relevant host group however, namely the TB-susceptible domestic animals other than cattle, is often neglected regarding TB control. Several of the above-mentioned tools can possibly be applied to more than one host category (Fig. 4).

The cattle TB control toolbox includes the well-established test and cull, but also the equally valuable farm biosafety and vaccination. These emerging tools deserve short comments as they are expected to grow in relevance over this decade.

Farm biosafety consists of the management and physical measures designed to reduce the risk of introduction, establishment and spread of infections (biosecurity measures, BSMs). It is a key disease control tool, especially where no vaccine is available nor authorized and when multi-host communities increase the risks, like for animal TB [59]. Biosecurity challenges vary greatly among production systems, and their management is influenced by numerous geographic, environmental, economic, socio-cultural, and political factors. In Mediterranean beef cattle farms, BSMs such as segregating wildlife and cattle at waterholes had an effect in terms of reducing cattle TB incidence [60]. Of the potential risk points, waterers, springs, and waterholes were the most common ones, and BSMs related to water management were identified as essential on most farms, along with BSMs regarding wildlife management. Farmers prioritized low-cost BSMs but 75% of the farmers adopted the proposed plans to some extent indicating that on-farm risk mitigation protocols are practical and feasible [33]. Spain, where wildlife is regionally regarded as an important barrier to TB control in cattle, has regulated wildlife management and biosafety in game species in an attempt to lessen this risk (https://www.boe.es/buscar/doc.php?id=BOE-A-2020-2109).

Regarding vaccination, the last 10 years have witnessed enormous progress in wildlife, cattle, and livestock other than cattle. Badger vaccination with BCG has been proven to contribute to TB control under field conditions and thus badger vaccination is now integrated into national control programs in the EU Member State Ireland and in the UK [61–64]. The effectiveness of cattle vaccination with BCG was recently reviewed and several new field trials were published, indicating a 28–85% efficacy under field conditions [65–67] and estimating that, in situations of low to moderate prevalence (< 15%), using BCG could lead to an OTF situation in 10 years [67]. In the UK, cattle vaccination with BCG is expected to become integrated into the national TB control program after developing a DIVA test and gaining WOAH acceptance to support the UK’s trade agreements [68].

Heat-inactivated mycobacteria are safe, have less logistic constraints than live vaccines, and are already used in commercial paratuberculosis (Johne’s disease) vaccines such as Silirum™ (CZ Vaccines, Porriño, Spain). Heat inactivated *M. bovis* is based on the same principle and has been shown to produce immunity against MTC in wild boar and domestic pigs [69–71], badgers [72], goats [73], red deer [74], cattle [75], zebrafish [76, 77], and African buffalo (*Syncerus caffir*) (Prof. Anita Michel, personal communication). Furthermore, heat inactivated mycobacteria confer cross-protection against a broad range of pathogens including *Salmonella* in pigs [78]. Two successful field trials have been run in wild boar [70, 79], but no licensing has taken place so far. Protective mechanisms have been associated with activation of innate immune response through the complement C3 pathway in response to biomolecules such as mycobacterial proteins and glycans such as Galα1-3Galβ1-(3)4GlcNAc-R (α-Gal) present in glycoproteins and glycolipids [80]. Furthermore, the immune response to heat-inactivated *M. bovis* has translated into adjuvant applications to boost immune response for the control of cattle tick infestations [81, 82].

The challenge for animal TB control policies is not only the uncertainty associated with epidemiological mechanisms driving the disease process, but also the geographical and temporal variation in public and stakeholder perception about the acceptability and effectiveness of the available control options [83, 84]. Worldwide, changes in the human demographic characteristics and social attitudes are underway, which are likely to force a critical reevaluation of the TB control strategies [85]. The success of any intervention is predicated on the ability and willingness of relevant human actors to routinely implement them. This might be shaped by the perceptions and subjective experiences of farmers and other actors. Standard test and cull disease control strategies, for instance, have significant impacts on the sustainability of farming, especially in small holdings [86] and this can affect farmer attitudes towards disease control. In the worst-case scenario, the distrust can permeate politics and hinder the continuation of successful schemes. Social science insights are needed to understand how individual, systemic, and more-than-human factors intersect and influence one another and are crucial to effectively engage all relevant stakeholders [39, 87]. In this regard, New Zealand is a showcase where livestock farmers are effectively involved in the national animal TB control strategy through the Animal Health Board. This kind of public-private partnership approach could be further explored to strengthen animal disease control.

In most parts of the globe, a complete eradication of *M. bovis* is unlikely to be achieved in the short or medium term. Even worse, at this global scale, animal TB prevalence figures are not improving recently for any of the three host categories except for very specific settings. This causes significant and prolonged costs and creates increasing distrust among the farmers and the field veterinarians about the practicality of ongoing TB control schemes. Hence, if the scientific community and the animal health managers want to make significant progress, we need to go beyond the paved roads.

**Fig. 5:**
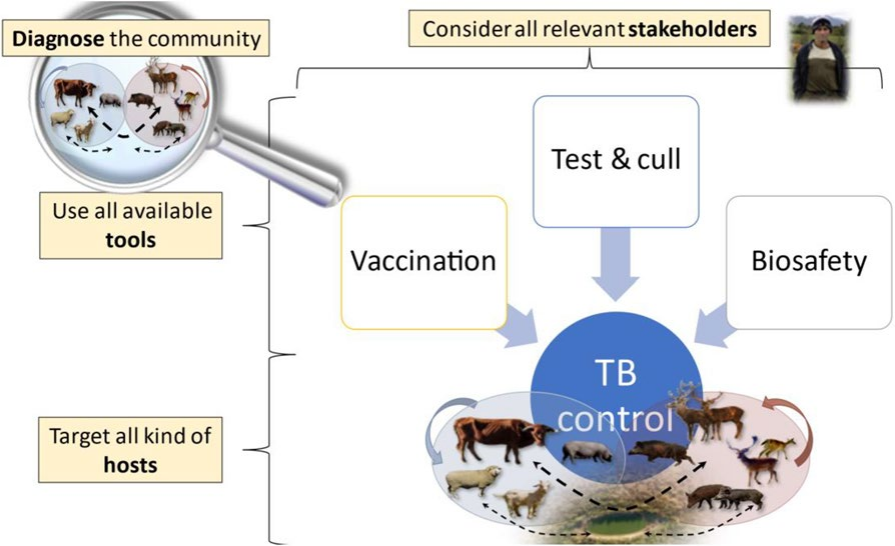
A schematic vision of integrated animal tuberculosis control. After defining the involved host species and their (environment mediated) interactions, an integrated scheme should target all relevant hosts and make use of all available control tools while involving the farmers and other relevant actors in decision making

In front of increasingly complex epidemiological scenarios, successful animal TB control requires first and foremost a clear vision and objectives and the willingness to hold firm [2]. Once the vision is clear, the starting point is a comprehensive assessment of the involved host species, environmental factors, and social context: the epidemiological diagnosis [31]. One example (in Spanish) is the Plan de Actuación sobre TUBerculosis en Especies Silvestres (PATUBES, https://www.mapa.gob.es/es/ganaderia/temas/sanidad-animal-higiene-ganadera/patubes2017_3_tcm30-378321.pdf). Existing programmes in Ireland and New Zealand represent ongoing examples of multiple host management when attempting to control animal TB. After this comprehensive assessment of the epidemiological situation, an integrated disease control scheme can be set up based on three pillars: (1) a close involvement of the farmers including collaborative decision making, (2) expanding the control target to the environment and all three host categories and their interactions, and (3) upgrading established control schemes from single-tool to integrated ones including farm biosafety and the strategic use of vaccination along with bolstering the existing test and cull schemes (Fig. 5).

## Data Availability

Not applicable.

## Abbreviations

AI: Artificial intelligence
BCG: Bacille Calmette Guerin
BSM: Biosecurity measure
bTB: Bovine tuberculosis
DIVA: Differentiating infected from vaccinated animals
e-DNA: Environmental DNA
LMIC: Low- and middle-income countries
*M. bovis*: *Mycobacterium bovis*
ML: Machine learning
MTC: *Mycobacterium tuberculosis* complex
OTF: Officially Tuberculosis Free
PATUBES: Plan de Actuación sobre TUBerculosis en Especies Silvestres
TB: Tuberculosis
WGS: Whole genome sequencing
WOAH: World Organization for Animal Health
